# Factors associated with the timing of antenatal clinic attendance among first-time mothers in rural southern Ghana

**DOI:** 10.1186/s12884-020-2738-0

**Published:** 2020-01-20

**Authors:** Alfred Kwesi Manyeh, Alberta Amu, John Williams, Margaret Gyapong

**Affiliations:** 1 Dodowa Health Research Centre, Dodowa, Ghana; 2 Division of Epidemiology and Biostatistics, School of Public Health, University of the Witwatersrand, Parktown, Johannesburg, South Africa; 3 Centre for Health Policy and Implementation Research, Institute for Health Research, University of Health and Allied Sciences, Ho, Volta Region Ghana; 4 INDEPTH Network, Accra, Ghana; 5 Ghana Health Service, Accra, Ghana

**Keywords:** Antenatal care, First-time mothers, Health and demographic surveillance system, Dodowa, Ghana

## Abstract

**Background:**

Pregnancy is an important period to promote healthy behaviors, prevent and identify diseases early and treat them to maximize the health and development of both the woman and her unborn child. A new World Health Organization antenatal care model recommends the initiation of antenatal care visit within the first trimester of gestation.

This study sought to examine the timing of initiation of antenatal care among first-time mothers and associated factors in rural Southern Ghana.

**Methods:**

Information on gestational age, timing of antenatal care, demographic and socioeconomic status of 1076 first-time mothers who gave birth in 2011 to 2013 in the Dodowa Health and Demographic Surveillance System were included in the study. The time of initiation of antenatal clinic attendance was calculated. The associations between dependent and independent variables were explored using logistic regression at 95% confidence interval in STATA version 14.2.

**Results:**

The mean gestational age at which the first-time mothers initiated antenatal care attendance was 3 month. Maternal age, level of education and household socioeconomic status were statistically significantly associated with timing of initiation of antenatal care attendance.

**Conclusion:**

Although more than half of the study participants initiated ANC visit in the first trimester of pregnancy, a high proportion also started ANC attendance after the World Health Organization recommended period. Maternal age is significantly associated with timing of initiation of antenatal care visit among first-time mothers; older women were more likely to initiate antenatal care visit in the first trimester of gestation compared to the younger women.

## Background

Pregnancy is an important time to encourage, allay anxieties and equip mothers with information which promotes healthy behaviors and parenting skills [1]. Appropriate care during pregnancy and childbirth is critical for the health of both the mother and the baby [2].

World Health Organization (WHO) recommends Antenatal Care (ANC) and suggests at least eight ANC visits in total, with the first visit during the first trimester of gestation [3, 4].

ANC is special care for pregnant women and it is important in the life of a pregnant woman and her family. It is a public health service with the goal of preventing health risks, early detection of abnormalities, institution of corrective measures if possible and preparation of both the woman and fetus and to ensure good start of life for each newborn child [5–7].

Suitable ANC introduces the pregnant woman to the health system. This enhances the probability of the woman birthing with a skilled birth attendant and contributes to the good health of both the mother and baby [1]. Insufficient ANC during pregnancy does not support the model of continuum of care, which might affect both mothers and babies [1].

While preparing for a safe childbirth is an essential part of ANC, the timely initiation of the first ANC visit is an important element [6, 8]. According to WHO, every pregnant woman in developing countries should seek ANC within the first trimester of gestation [9, 10]. WHO guidance focuses on; preventing and treatment of anaemia by encouraging the pregnant woman to take iron and folate supplements, prophylactic treatment of malaria, immunization against tetanus, tuberculosis (TB).

Health education on nutrition, monitoring and treatment of sexually transmitted infections (STIs) including human immune virus/acquired immune deficiency syndrome (HIV/AIDS) as well as early detection and management of other chronic diseases and warning signs of complications is also achieved during this period [9, 10].

Studies have shown that, early ANC attendance (during the first trimester of pregnancy) plays a major role in early detection and treatment of maternal health problems inpregnancy and serves as a good basis for proper management during and after childbirth [6, 8]. Hence, failure to initiate ANC early is a potential risk for complications during pregnancy, childbirth, and puerperium [6, 8].

Late initiation of ANC may lead to late diagnosis of complications which might have the potential to detrimentally affect maternal and fetus health. Thus, contributes to maternal mortality, premature labour, preterm babies and intra-uterine deaths [11]. In Ghana ANC forms the basis of all maternal health care provision, and encompasses the evaluation of the general health of pregnant women with the goal of detecting and preventing adverse maternal and neonatal outcomes. ANC in Ghana, is provided by qualified health-care professionals (Doctors, Nurse, Midwives, and Community Health Nurses) [12, 13].

ANC conventionally takes the form of a one-on-one consultation between a pregnant woman and her health-care provider. The antenatal visit in Ghana integrates the usual individual pregnancy health assessment with tailored group educational activities and peer support, with the aim of motivating behaviour change among pregnant women, improving pregnancy outcomes, and increasing women’s satisfaction.

The 2014 Ghana Demographic and Health Survey (GDHS) showed that 97% of females who gave birth in the 5 years preceding the survey received ANC at least once for their last childbirth and approximately nine in ten women had four or more ANC visits [14].

Studies elsewhere have identified several factors such as media exposure, maternal education, health service availability, husband’s education, cost, household income, history of obstetric complications and women’s employment that impact on use of ANC, in developing countries [15–18]. These studies are supported by other research finding late ANC attendance is associated with young maternal age, lack of partner or family support, high parity, premarital status, lack of formal education, unwanted pregnancies and low socioeconomic status [17–19].

In Ghana, there is a lack of research as to the factors affecting timing of initiation of ANC attendance. Hence this study examines timing of initiation of ANC attendance and associated factors among first-time mothers in rural Southern Ghana.

## Methods

### Study area

The republic of Ghana is located on the West African Coast and the study was conducted in two rural districts. The Shai-Osudoku and Ningo-Prampram districts of the Greater Accra Region of Ghana have a total population of 115,7754. A detailed description of the Dodowa Health and Demographic Surveillance System (DHDS and its operations can be found elsewhere [20–22]. The study used secondary data from DHDSS.

### Study participants

The target population was made up of first-time mothers who were resident in DHDSS and had their first birth from 2011 to 2013. All women who were not first-time mothers, who were not captured in the DHDSS and who gave birth before 2011 or after 2013 were excluded from the study. All participants included in the study aged 17 years and above.

### Variables

The dependent variable for this study is timing of ANC visit which was recorded as: 1 “Within first trimester”, 0 “After first trimester”. From the available DHDSS data, we extracted 7 independent variables which were based on literature and the likelihood to influence the outcome of interest. These independent variables include: maternal age, education, marital status, household size, household head’s education, district and socio economic status.

Determination of socioeconomic status as a proxy measure of a household’s long term standard of living using calculated weights based on principal component analysis (PCA) [23] has been reported elsewhere [20, 24].

### Statistical methods

The sociodemographic data for all women who met the inclusion criteria was extracted from the longitudinal population-based electronic database of the DHDSS. The extracted data was exported to STATA version 14.2 for cleaning, coding and analysis. A descriptive analysis of socio-demographic characteristics of the participants was conducted. The associations between the independent variables and the outcome of interest were examined in unadjusted and adjusted logistics regression model. The exposure variables that were significant at *p* < 0.05 in the unadjusted model were entered together into an adjusted model. Version 14.2 of Stata was used for the data analysis and the results were presented in charts and tables with summary statistics in odds ratios (OR), with 95% confidence intervals (CI) and *p*-values.

## Results

### Socio demographic and pregnancy related characteristics of the study participants

**Fig. 1:**
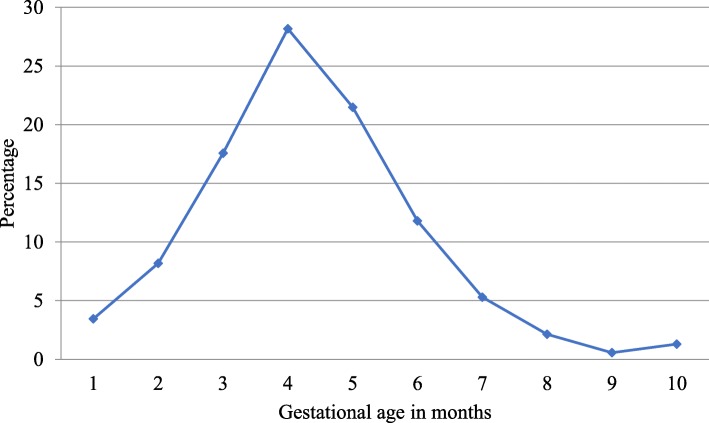
Percentage of first-time mothers by gestational age at ANC visit initiation

The study includes a total of 1076 first-time mothers whose socio-demographic characteristics are presented in Table 1. The average gestational age at which the study participants initiated ANC visit was 3.4 months. The detailed distribution of gestational age of study participants at initiation of ANC attendance is shown in Fig. 1. While most of the first-time mothers initiated ANC visit in the first trimester of gestation (57%), 39 and 4% initiated ANC visit in the second and third trimesters respectively.

**Table 1:** Socio-Demographic Characteristics of the study participants

Characteristics	Frequency (*n* = 1076)	Proportion (%)
Age Group		
< 20	345	32
20–24	350	33
25–29	220	20
30 +	161	15
Ethnicity		
Ga-Dangme	777	72
Other Tribes	299	28
Religion		
Christianity	1003	93
Other Religions	73	7
Occupation		
Unemployed	237	22
Farmer	106	10
Artisan	118	11
Trader	209	19
Civil Servant	27	3
Student	358	33
Others	21	2
Education		
Primary / No Education	590	55
Junior High School	357	33
Senior School Level	129	12
Marital Status		
Never married	676	64
Ever married	378	36
Timing of ANC visit		
After first trimester	459	43
Within first trimester	617	57
Mean gestational age (in months) = 3.36		
Household size		
Five or less	529	49
More than five	547	51
Mean household size = 6.52 (SD = 4.73)		
Household head’s Education		
No education / primary	564	52
JHS and above	512	48
District		
Shai-Osudoku	508	47
Ningo-Prampram	568	53

The average age was 23 years. A higher proportion of the study participants (72%) were of the Ga-Dangme ethnic group and 93% were Christians. While 33% of study participants were students, 22 and 19% of them were unemployed and traders respectively. While 39% of the respondents had primary level of education, 33 and 12.0% were educated up to junior and senior high levels of education respectively. About 16% of the participants had no formal education. More than half of the study participants (64%) said they were never married.

### Unadjusted and adjusted odds of determinants of ANC initiation among first-time mothers

In both the unadjusted and adjusted logistic models as shown in Table 2, the odds of first-time mothers initiating ANC visit in the first trimester of gestation increased with increasing maternal age. In the unadjusted model, the odds of first-time mothers initiating ANC visit in the first trimester of gestation was 82% higher for mothers aged 20-24 years compared to those aged < 20 years (OR: 1.82, 95%CI: 1.34–2.46). Mothers aged 25–29 and 30+ years in the unadjusted model were more than twice likely to initiate ANC attendance in the first trimester compared to those aged < 20 years. Age 25-29 years (OR: 2.22, 95%CI: 1.57–3.15) and age 30 + years (OR:2.30, 95%CI: 1.56–3.40).

**Table 2:** Unadjusted and adjusted odd ratios of determinates of initiation of ANC attendance among first time mothers

	Unadjusted		Adjusted^b^	
Characteristics	OR	*P*-Values (95% CI)	OR	*P*-Value (95% CI)
Maternal Age/year				
< 20	1.00		1.00	
20–24	1.82(1.34–2.46)^a^	< 0.001	1.62(1.18–2.24)^a^	0.003
25–29	2.22(1.57–3.15)^a^	< 0.001	1.64(1.08–2.48)^a^	0.019
30+	2.30 (1.56–3.40)^a^	< 0.001	1.80(1.15–2.82)^a^	0.010
Mothers Occupation				
Unemployed	1.00		1.00	
Farmer	1.28(0.80–2.05)	0.296	1.27(0.78–2.06)	0.330
Artisan	1.58(1.00–2.50)	0.052	1.24(0.76–2.00)	0.388
Trader	1.13(0.78–1.65)	0.510	1.06(0.72–1.58)	0.761
Civil Servant	3.56(1.30–9.72)^a^	0.013	1.87(0.62–5.60)	0.264
Student	0.86(0.62–1.19)	0.354	1.00(0.70–1.42)	0.999
Others	2.02(0.76–5.39)	0.159	2.03(0.74–5.58)	0.170
Maternal Education				
No Education / Primary	1.00		1.00	
JHS Level	1.36(1.04–1.78)^a^	0.023	1.04(0.76–1.41)	0.729
SHS and above	2.44(1.60–3.72)^a^	< 0.001	1.49(0.90–2.46)	0.307
Marital Status				
Never married	1.00			
Ever married	1.14(0.88–1.47)	0.319		
Socio Economic Status				
Poorest	1.00		1.00	
Poorer	0.81(0.55–1.18)	0.270	0.94(0.63–1.39)	0.744
Poor	1.24(0.84–1.81)	0.278	1.32(0.89–2.00)	0.162
Less Poor	0.96(0.66–1.41)	0.853	1.03(0.70–1.51)	0.898
Least Poor	1.78(1.20–2.63)^a^	0.004	1.43(0.95–2.17)	0.087
Household Head Level of education				
No Education / Primary	1.00		1.00	
JHS and above	1.43(1.12–1.82)^a^	0.004	1.09(0.83–1.44)	0.519
District				
Shai-Osudoku	1.00			
Ningo-Prampram	0.96(0.75–1.22)	0.739		

*OR* Odd Ratio, *CI* Confidence Interval, *SD* Standard deviation, ^a^statistically significant, ^b^Correct classification rate of the model = 59.94%

After adjusting for maternal occupation, maternal education, socioeconomic status and household head’s level of education, there was an increasing odds of 62, 64 and 80% for maternal age 20-24 years, 25-29 years, and 30 + years respectively for first-time mothers to initiate ANC visit in first trimester of pregnancy compared to mothers aged < 20 years (OR: 1.62, 95%CI: 1.18–2.24, OR:1.64, 95%CI: 1.08–2.48 and OR: 1.80, 95%CI: 1.15–2.82) respectively.

Although farmers, Artisans, and Traders had an increased odds of 28, 58 and 13% respectively of initiating ANC visit in the first trimester of pregnancy compared to those unemployed (OR:1.28, 95%CI: 0.8–2.05, OR:1.58, 95%CI:1.00–2.50, OR:1.13, 95%CI:0.78–1.65). Civil servants were more than thrice likely to initiate ANC visit in the first trimester of pregnancy as compared to those unemployed (OR: 3.56, 95% CI: 1.30–9.72) in the unadjusted model.

After adjusting for other explanatory variables, there was an increased odds of 27, 24, 6 and 87% of Farmers, Artisans, Traders and Civil Servants respectively initiating ANC visit in the first trimester of pregnancy (OR: 1.27, 95%CI:0.78–2.06, OR:1.24, 95%CI:0.76–2.00, OR:1.87, 95%CI:0.62–5.60). Increasing level of education was statistically significantly associated with timing of initiating of ANC visit in the unadjusted model such that participants who had Junior High School (JHS) level of education were 36% more likely to initiate ANC visit in the first trimester compared to those with no education (OR: 1.36, CI: 1.04–1.78) and mothers with Senior High School (SHS) and above level of education are more than twice more likely to initiate ANC visit in the first trimester compared to those with no education (OR:2.44, 95%CI:1.60–3.72).

Although there was an increased odds of 4 and 49% for mothers with JHS, SHS and above level of education to initiate ANC visit in the first trimester after adjusting for other explanatory variables, this was not statistically significant (OR:1.04, 95%CI:0.76–1.41, OR:1.49, 95%CI:0.90–2.46). There was an increased odds of first-time mothers who were ever married to initiate ANC visit in the first trimester (OR: 1.14, 95%CI: 0.88–1.47).

Participants who belong to the richest socioeconomic status were 78% more likely to initiate ANC visit in the first trimester of pregnancy in the unadjusted model (OR:1.78, 95%CI:1.20–2.63). After adjusting for other explanatory variables, there was an increased odds 43% of women who belong to the richest socioeconomic status to initiate ANC visit in the first trimester (OR:1.43, 95%CI: 0.95–2.17).

First-time mothers whose heads of household have JHS and above level of education were 43% more likely to start ANC visit in the first trimester (OR: 1.43, 95%CI:1.12–1.82) but this was not statistically significant in the adjusted model (OR:1.09, 95%CI:0.83–1.44).

## Discussion

The distribution of the socio-demographic characteristics of the study participants is comparable to the findings of earlier studies in the two study districts [20, 22, 24, 25].

The World Health Organization (WHO) recommend that, a pregnant woman needs to initiate antenatal care in the first trimester of pregnancy. However, a significant proportion of women from developing countries do not adhere to the WHO recommendation [26]. This study revealed that 57% of the pregnant women initiated ANC visit within the first trimester of gestation. This finding is higher than what was found in other studies in sub-Saharan Africa [27–30] but lower than what is recommended by WHO [3, 31]. This divergence could be due to the economic, socio-cultural and timing differences between the studies and among the study population as suggested in other studies [32, 33]. The late ANC attendance may prevent women from having the full benefit of preventive and early disease detection and treatment strategies, such as the use of iron and folate supplements for the treatment of anaemia, prevention of malaria in pregnancy through administration of Intermittent Preventive Treatment (IPTp), immunization against tetanus, TB, nutrition, and detection and management of HIV/AIDS and other STIs [9, 10]. Hence, it is likely that some of these first-time mothers missed critical services offered during the first trimester ANC visit such as risk screening, preventive health measures and health education.

Our study revealed that majority of the study participants had no education or only attained primary education which is also consistent with findings of Gebremeske et al. [2]. This study also showed that the mean gestational age at which first-time women initiate ANC visit is 3 months. This is lower than what has been found in a similar study in Ethiopia [2]. This may be due to the high coverage of ANC attendance in Ghana as reported by Ghana Statistical Services [14, 34].

In this study, first-time mothers younger than 20 years were less likely to initiate ANC visit within the WHO recommended time (within first trimester of pregnancy) compared to those who were older. This finding supports the results of a study conducted in Ghana, Kenya and Malawi [35] which suggested that due to social ramification of teenage pregnancy which includes dismissal from school and stigma, adolescents are at risk of hesitating pregnancy disclosure and therefore ANC attendance. The finding of this study is consistent with the findings of studies conducted in Nigeria and Ethiopia which suggested that women with intended pregnancy were more likely to initiate ANC attendance earlier compared to unintended ones [36, 37]. This finding is consistent with a study conducted in Addis Ababa [37]. If a pregnancy is planned, women might be prepared to initiate ANC early as shown in a study [38]. It is believed that intended pregnancies are more cared for by pregnant women and their partners; this enabling factor for women to initiate ANC timely. In Ghana, very few studies have been conducted on unintended pregnancies. This presents the need for further investigation in the Ghana context.

### Strengths and limitations

There is little evidence on coverage of early initiation of antenatal care visits globally [4]. Hence, the large sample size, data quality, population based nature and focus on rural communities which are priority for public health interventions is a major strength of the study. This notwithstanding, the study had a number of shortcomings and limitations. The secondary data used did not include other important variables such as; type and doses of treatments received during ANC visits and evidence of services provided at each ANC visit. The data used was also not a nationally representative one. Since the two study districts cannot be true representative of 216 districts in Ghana, the findings cannot be generalized to the whole country.

## Conclusions

In this study, we have shown that less than half of the study participants initiated ANC visit after first trimester of pregnancy which is outside the gestation recommended by the WHO. Investment in communicating strategies to target young women and families prior to pregnancy about timely ANC visiting expectations as a health priority will likely be of greatest benefit. This is recommended by the new WHO model for women in rural settings [3].

Furthermore, a context specific implementation research is needed to better understand the reasons why pregnant women might not initiate antenatal care in the first trimester of pregnancy and to address these gaps using innovative interventions that are appropriate to local settings as recommended by the 2016 WHO ANC guideline [3].

## Data Availability

We are unable to share the data used for this study publicly due to the ethical policies, and the data sharing agreement of DHRC. Nevertheless, the data is available upon request through the corresponding author subject to the data sharing policy of DHRC.

## Abbreviations

ANC: Antenatal care
CI: Confidence intervals
DHDSS: Dodowa Health and Demographic Surveillance System
DHRC: Dodowa Health Research Centre
GDHS: Ghana Demographic Health Survey
HIV/AIDS: Human immune virus/acquired immune deficiency syndrome
IPTp: Intermittent Preventive Treatment
MDG: Millennium Development Goal
MICS: Multiple Indicator Cluster Survey
MMR: Maternal Mortality Ratio
OR: Odd ratio
PCA: Principal component analysis
SDG: Sustainable Development Goal
STI: Sexually transmitted infections
TB: Tuberculosis
WHO: World Health Organization
